# Supplementary material for: Evaluation of longitudinal time-lapsed in vivo micro-CT for monitoring fracture healing in mouse femur defect models
Source: Sci Rep. 2019 Nov 25;9:17445. doi: 10.1038/s41598-019-53822-x (PMC6877534; doi:10.1038/s41598-019-53822-x)
Supplement: Supplementary file 1 — Supplementary Information [file 41598_2019_53822_MOESM1_ESM.pdf]

Evaluation of longitudinal time-lapsed *in vivo* micro-CT for monitoring fracture healing in mouse femur defect models

Esther Wehrle[1], Duncan C Tourolle né Betts[1], Gisela A Kuhn[1], Ariane C Scheuren[1], Sandra Hofmann[1,2], Ralph Müller[1*]

[1] Institute for Biomechanics, ETH Zurich, Zurich, Switzerland, [2] Department of Biomedical Engineering and Institute for Complex Molecular Systems, Eindhoven University of Technology, The Netherlands.

Corresponding author:

Ralph Müller, PhD

Institute for Biomechanics

ETH Zurich

Leopold-Ruzicka-Weg 4

8093 Zurich, Switzerland

Email: ram@ethz.ch

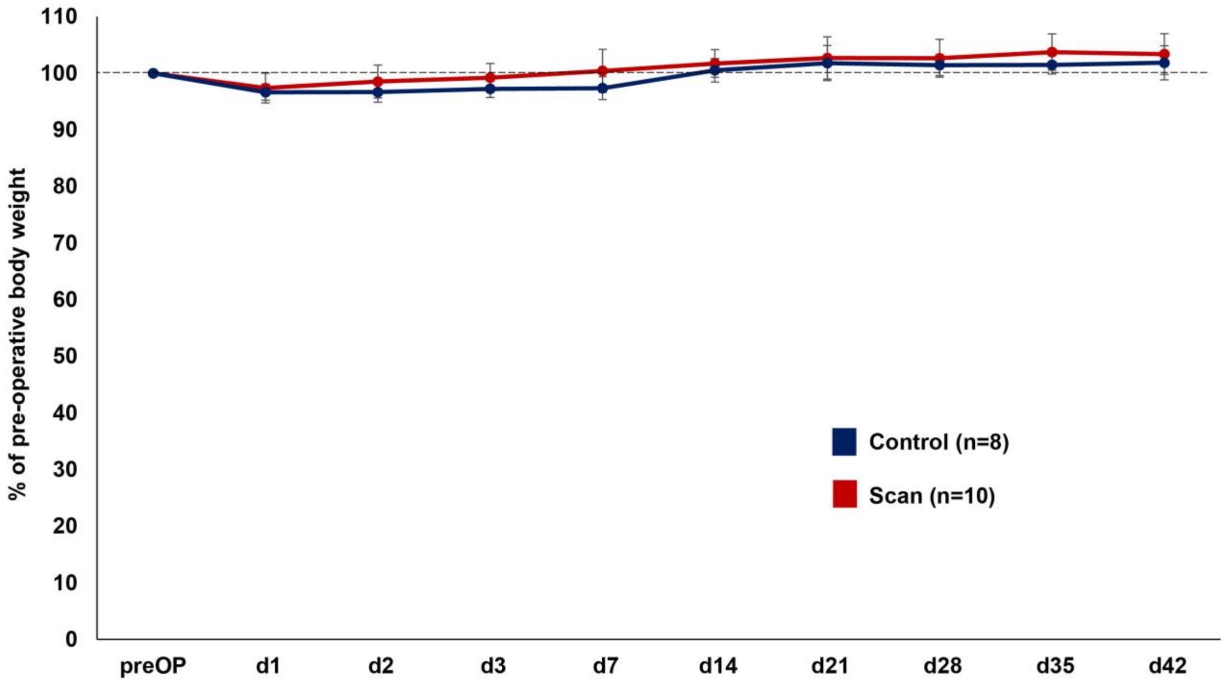

Supplementary Fig. S1. *In vivo* monitoring of body weight of the mice from the control group (n=8) and the scan group (n=10) measured pre-operatively (preOP), on postoperative days 1-3 and weekly from day 7 to day 42. The postoperative values were related to the preoperative data.

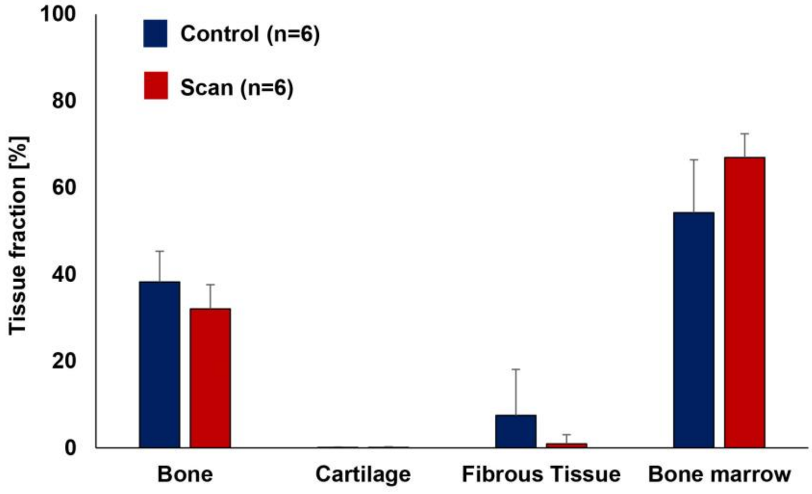

Supplementary Fig. S2. Callus composition on day 42 as assessed by histomorphometry on Safranin-O stained tissue sections in animals from the control group (n=6) and the scan group (n=6).

Supplementary Table S1. Study design (female 20 week-old C57BL/6J mice)

Group	Group size	Femur defect (at 20 weeks of age)	*In vivo* micro-CT measurements	Registration of micro-CT scans #	Histology
Scan	n=11	x (n=11)	d0, week 1-6 (n=11)	week 1-6 to week 0-5 (n=10)	week 6 (n=6)
Control	n=8	x (n=8)	d0, week 5+6 (n=8)	week 6 to week 5 (n=8)	week 6 (n=6)

micro-CT scan taken at timepoint x registered to micro-CT scan taken at timepoint x-1
